# Biodiversity Genomics Research Practices Require Harmonising to Meet Stakeholder Needs in Conservation

**DOI:** 10.1111/mec.70001

**Published:** 2025-06-26

**Authors:** Elena Buzan, Christian de Guttry, Chiara Bortoluzzi, Nathaniel R. Street, Kay Lucek, Anna Rosling, Lino Ometto, Alice Mouton, Luísa S. Marins, María José Ruiz‐López, José Melo‐Ferreira, Elisabet Ottosson, Camila J. Mazzoni, Robert M. Waterhouse

**Affiliations:** ^1^ Faculty of Mathematics, Natural Sciences and Information Technologies University of Primorska Koper Slovenia; ^2^ Faculty of Environmental Protection Velenje Slovenia; ^3^ SIB Swiss Institute of Bioinformatics, Environmental Bioinformatics Group Amphipôle, Quartier UNIL‐Sorge Lausanne Switzerland; ^4^ Department of Biology University of Florence Florence Italy; ^5^ Umeå Plant Science Centre, Department of Plant Physiology Umeå University Umeå Sweden; ^6^ SciLifeLab, Department of Plant Physiology Umeå University Umeå Sweden; ^7^ Biodiversity Genomics Laboratory, Institute of Biology University of Neuchâtel Neuchâtel Switzerland; ^8^ Department of Ecology and Genetics Uppsala University Uppsala Sweden; ^9^ Department of Biology and Biotechnology University of Pavia Pavia Italy; ^10^ National Future Biodiversity Center (NFBC) Palermo Italy; ^11^ Département Sciences et Gestion Environnement, SEED University of Liège Arlon Campus Arlon Belgium; ^12^ Berlin Center for Genomics in Biodiversity Research (BeGenDiv) Berlin Germany; ^13^ Evolutionary Genetics Department Leibniz‐Institut für Zoo‐ Und Wildtierforschung (IZW) Berlin Germany; ^14^ Departamento de Biología de la Conservación y Cambio Global Estación Biológica de Doñana (EBD), CSIC Sevilla Spain; ^15^ CIBER de Epidemiología y Salud Pública (CIBERESP) Madrid Spain; ^16^ CIBIO, Centro de Investigação Em Biodiversidade e Recursos Genéticos, InBIO Laboratório Associado Universidade Do Porto Vairão Portugal; ^17^ Departamento de Biologia, Faculdade de Ciências Universidade do Porto Porto Portugal; ^18^ BIOPOLIS Program in Genomics Biodiversity and Land Planning, CIBIO, Campus de Vairão Vairão Portugal; ^19^ SLU Swedish Species Information Centre Swedish University of Agricultural Sciences Uppsala Sweden

**Keywords:** best practices, biodiversity genomics, genome‐wide genetic diversity, stakeholder engagement, standardisation, whole genome resequencing data

## Abstract

Biodiversity resilience relies on genetic diversity, which sustains the evolutionary potential of organisms in dynamic ecosystems. Genomics is a powerful tool for accurately estimating genetic diversity across genomes of species and populations. However, integration of genomic data into conservation efforts faces challenges due to the heterogeneity of approaches employed. Establishing common sets of standards for genomic data production and analysis is essential to consistently interpret results and clearly communicate outcomes to stakeholders. While the European Reference Genome Atlas (ERGA) community has contributed significantly to the standardisation of reference genome methodologies in synergy with other initiatives, there is now an urgent need to extend these principles to downstream analyses. ERGA aims to build on its experience to help establish harmonised approaches in applied biodiversity genomics research, aligned with ongoing efforts to define standardised metrics for measuring and reporting genetic diversity. Establishing consensus on best practices for genome‐wide data generation methods and applications will substantially increase accuracy, interpretability, and comparability, together with enhanced stakeholder capacities. By identifying key opportunities and challenges, as well as conducting preliminary stakeholder mapping and examining case studies, the goal is to build an inclusive framework that ensures the relevance and widespread adoption of these best practices: fostering trust and confidence in genomics research practices to meet stakeholder needs in biodiversity conservation. We call upon the broader research community to join efforts in establishing these approaches, recognising the importance of participation of end‐users, to foster the integration of genomic data into the toolkit for measuring and reporting genetic diversity.

## 1 Introduction

Genetic diversity is fundamental to species resilience and adaptability, enabling organisms to respond to environmental changes and anthropogenic pressures and determining their evolutionary potential for long‐term survival within dynamic ecosystems (Aitken et al. 2024; Kardos and Luikart 2021). Assessing genetic diversity is thus essential for developing informed biodiversity conservation and management strategies. While various approaches exist for evaluating genetic diversity, achieving a comprehensive overview with high accuracy, precision, and comparability relies on genome‐wide data analysis at the population level (Kardos et al. 2021). Combining reference‐quality genome assemblies with whole‐genome re‐sequencing (WGS) data (see Glossary), referred to as the assessment of genome‐wide genetic diversity (see Glossary), greatly enhances the resolution and reliability of evidence available for practical biodiversity conservation actions (Fuentes‐Pardo and Ruzzante 2017; Hogg 2024; Jeon et al. 2024). Applied genome‐wide genetic diversity assessments are increasingly being employed to inform conservation of endangered species such as the Tasmanian devil (Wright et al. 2020), the kākāpō (Dussex et al. 2021), the Yangbi County maple (Ma et al. 2022), killer whales (Kardos et al. 2023), blue whales (Wolf et al. 2024), the Chinese pangolin (Wei et al. 2024), or the North Atlantic right whale (Orton et al. 2024), and in pioneering regional programmes like the California Conservation Genomics Project (see Glossary) (Shaffer et al. 2022). Although the advance of genomics technologies has revolutionised practices relying on genetic data, scientific community consensus on data production and analysis methods is needed for applied population genomics studies to deliver results that can meaningfully inform conservation actions and species genetic composition monitoring.

Building consensus within the scientific community on genomics research standards and best practices is an important first step towards successful stakeholder engagement and the increasingly widespread adoption of genomic data (Field et al. 2011). Promoting such harmonised and validated methodologies will foster trust and confidence in genomics research practices, thereby advancing their acceptance and application by stakeholders (Bateman and Balmford 2023; Pärli et al. 2021; Supple and Shapiro 2018). To begin to address these issues, we present current perspectives on the need for standardisation and harmonisation (see Glossary) in genome‐wide genetic diversity assessments and issue a call to the scientific community to collaborate on this timely effort, recognising that it requires broad participation and consensus to overcome key challenges. The goal of co‐creating standards within the scientific community, with a focus on stakeholder needs, is to optimise the integration of genomic approaches into biodiversity conservation practices. This approach necessitates a transition from a narrow scientific perspective to a more stakeholder‐inclusive perspective, emphasising the understanding and prioritisation of stakeholder views. To initiate these efforts, we set out how consensus methods and standards can advance science and enhance confidence amongst user‐groups. We present first steps towards user engagement through stakeholder mapping exercises, and we examine case studies to gain practical insights into stakeholder engagement activities in biodiversity genomics. Through these collaborative endeavours, we strive to facilitate the building of community consensus on standards that align biodiversity genomics research practices with real‐world conservation demands and stakeholder needs.

## 2 Consensus Methods and Standards Advance Science and Enhance Confidence

### 2.1 Community Efforts on Standardised Information to Measure and Report Genetic Diversity

The importance of consensus is highlighted through ongoing global efforts to develop consistent information to measure and report genetic diversity. For example, as part of the Kunming‐Montreal Global Biodiversity Framework (KMGBF), the Convention on Biological Diversity (CBD) encourages the use of standardised Essential Biodiversity Variables (EBVs) (Pereira et al. 2013) developed by the Group on Earth Observations Biodiversity Observation Network (GEO BON) to help inform the indicators needed to monitor biodiversity change (COP 15 2022). Amongst the 23 KMGBF targets, target four explicitly highlights the importance of maintaining genetic diversity within and between populations for species adaptability and survival (COP 15 2022). To support this goal, three indicators were developed to measure genetic diversity between and within populations (Mastretta‐Yanes et al. 2024). Two of these were adopted by the CBD as headline and complementary indicators, respectively, the effective population size above 500 (*Ne* 500) indicator and the populations maintained (PM) indicator. Both the *Ne* 500 and PM indicators can be based on DNA data, whenever available, or alternative proxy‐based assessments (Hoban, Paz‐Vinas, et al. 2024). Within the CBD framework, different initiatives have supported the development and testing of genetic diversity indicators, as well as adapting concepts, protocols, and tools for the assessment, monitoring, and management of genetic diversity: these include the Society for Conservation Biology's Conservation Genetics Working Group, COST actions such as Genomic Biodiversity Knowledge for Resilient Ecosystems (G‐BiKE) and the Genetic Nature Observation and Action (GENOA), the International Union for Conservation of Nature Species Survival Commission's Conservation Genetics Specialist Group, and the GEO BON Genetic Composition Working Group, coordinating their efforts through the Coalition of Conservation Genetics (Kershaw et al. 2022). In addition to the CBD‐endorsed Biodiversity Indicators, the GEO BON Genetic Composition Working Group has defined genetic composition EBVs through a collaborative international effort and consensus‐building process (Hoban et al. 2022). This initiative established consensus metrics for monitoring within‐species genetic variation, including genetic diversity (richness and heterozygosity), genetic differentiation (number of genetic units and genetic distance amongst them), effective population size, and inbreeding. These and other efforts highlight the global recognition of the need for action and the importance of community consultation and consensus‐building to ensure the relevance and adoption of standardised information to measure and report genetic diversity.

While existing non‐DNA‐based information such as species occurrences and summaries of previous genetic datasets can be used to assess genetic status, trends, and/or the effectiveness of some conservation actions (Hoban, da Silva, et al. 2024; Hoban, Paz‐Vinas, et al. 2024), genomic data are essential for accurately estimating several key genetic composition parameters. These parameters are vital for understanding the genetic health and viability of populations (Jeon et al. 2024; Kurland et al. 2024; Lavanchy and Goudet 2023) and include effective population size (Ne) (see Glossary) and runs of homozygosity (RoH) (see Glossary). Ultimately, such estimates facilitate the assessment of inbreeding and genetic drift—critical factors in conservation and within‐species genetic monitoring, especially for small populations (Hoban, da Silva, et al. 2023; Hoban, Paz‐Vinas, et al. 2024; Jeon et al. 2024; Waples 2022). Genomic data also provide the information required to perform robust evaluations of local adaptation and population differentiation (Hoban et al. 2016; Lehnert et al. 2023), while offering valuable insights into complex variations, such as structural variants, thereby deepening our understanding of species' evolutionary dynamics (Lee et al. 2023; Wold et al. 2021). These and additional advantages of applying genome‐wide data to exploring genetic variation—encompassing both neutral and functional variation—or the factors that influence it are further detailed in recent community perspectives (e.g., Formenti et al. 2022; Hogg 2024; Kardos et al. 2021; Theissinger et al. 2023). The common theme is that genome‐wide data anchored on chromosome‐level reference genomes (see Glossary) can provide high‐quality, comparable and scalable estimates of genetic diversity metrics, including genetic composition EBVs, by capturing the complete genetic variation of species and populations. Therefore, community efforts on the standardisation of information supporting indicators and EBVs need to be extended to accelerate the integration of genomic data into the toolkit for measuring and reporting genetic diversity.

### 2.2 Reference Genomes as an Example of Standardisation in Genomics Science

Advances in sequencing technologies have revolutionised reference genome generation, leading to the shift from contig‐ or scaffold‐level assemblies to fully resolved chromosome‐level reference genomes, which have now become the standard to anchor studies on genome structure, species divergence, and population genetics (Blaxter et al. 2022; Li and Durbin 2024). International collaborative efforts, such as the Earth BioGenome Project (EBP) (see Glossary), exemplify how coordinated initiatives involving a diverse group of international experts can help the scientific community establish consensus standards and guidance for each stage of the reference genome production workflow—from sampling, sequencing and assembly to deposition in open international data repositories (Lawniczak et al. 2022; Lewin et al. 2018, 2022). Setting baseline quantitative assembly standards—such as an error rate below 1 in 10,000 basepairs and chromosome‐scale scaffolding–offers clear targets towards which the community can aim. Initiatives like the European Reference Genome Atlas (ERGA) (see Glossary) have demonstrated the practical importance of these standards in scaling up reference genome production on the European continent (Böhne et al. 2024; Mc Cartney et al. 2024). Importantly, the common goals of research excellence continue to encourage streamlining and standardisation of best practices, combinations of methods, and quality control procedures that mostly routinely enable the attainment of the high‐quality target standards. The consensus approach to harmonising research practices in reference genome generation demonstrates that creating and implementing standards is a collaborative effort that requires the involvement of the entire scientific community. By establishing consensus on methodologies and providing guidance through training platforms, the community enhances confidence in the data produced and promotes widespread adoption of these best practices. The reference genomes example illustrates how science standardisation can advance research and facilitate worldwide endeavours, here focused on promoting the scaled‐up production and use of chromosome‐scale reference genome assemblies.

### 2.3 Genetic Diversity Assessments Would Benefit From Enhanced Method Standardisation

Data and methods for genome‐wide genetic diversity assessments are currently in a phase akin to that of generating genome assemblies prior to the establishment of the EBP and ERGA, as they have yet to undergo similar methodological harmonisations. Genetic diversity assessment methods have evolved substantially since the introduction of allozymes in the late 1960s (Prakash et al. 1969), moving from being based on a few genetic loci to covering thousands or millions of markers across the whole genome (Allendorf 2017; McMahon et al. 2014; Supple and Shapiro 2018). Examples include techniques targeting subsets of the genome, such as medium‐ to high‐density single nucleotide polymorphism (SNP) panels or reduced representation sequencing like restriction site‐associated DNA (RAD) sequencing (Baird et al. 2008). Although widely used because of their relatively low cost, these sequencing methods provide only a partial view of genetic diversity within a population or species and are not designed to detect fine‐scale, genome‐wide genomic variation (Helyar et al. 2011; Kardos et al. 2021; Shafer et al. 2017). While technological advances have driven major improvements in both the quality and quantity of genetic data generation that have greatly enhanced our understanding of population genetics and enabled precise estimates of population health (Hogg et al. 2022; Supple and Shapiro 2018), they have also indirectly introduced challenges due to the employment of a large variety of laboratory protocols, targeted genomic regions, and analytical approaches (Cariou et al. 2016; Davey et al. 2011, 2013; Lavanchy and Goudet 2023).

Considering this heterogeneity, approaches employing WGS data offer important opportunities for achieving consensus standardisations that allow for detailed genome‐wide analyses of genetic variation and more robust comparisons across studies and over time (Hogg et al. 2022; Jeon et al. 2024; Kardos et al. 2021; Kurland et al. 2024; Shafer et al. 2015). Broadly speaking, WGS data can be categorised based on their sequencing coverage into two main types: low‐coverage (lcWGS, generally less than 10×) where individuals are sequenced at depths too low to confidently call genotypes, thereby creating the need to incorporate uncertainties into downstream analyses or consider populations rather than individuals as the analysis unit; and high‐coverage (hcWGS, generally 20–30×, but this may depend on the system and the goals) where sequencing depths allow confident genotype calling at most sites across the entire genome, including even high‐complexity regions such as the major histocompatibility complex (Silver et al. 2024). However, there is no simple answer as to where to draw the line, and the answer may vary depending on additional factors such as levels of linkage disequilibrium or the availability of high‐quality reference panels (Benjelloun et al. 2019). Indeed, intermediate sequence depths may often provide sufficient information to resolve targeted questions, but they can lead to certain limitations, e.g., confidently defining structural or copy number variations across individuals or investigating species where sex chromosome coverage would be halved in the heterogametic sex. Any distinction between lcWGS and hcWGS or the range between them becomes even more complex when dealing with species that have large repeat‐rich and/or polyploid genomes, where even high coverage may still fail to provide truly complete and high‐quality datasets. In many cases, lcWGS data may comprise an important first step towards more in‐depth analyses with higher‐coverage data, e.g., for testing workflows and determining coverage requirements for high‐quality inferences (Caccavo et al. 2024). Nevertheless, while acknowledging specific practical limitations, e.g., with large genomes where costs could become prohibitive, hcWGS data offer the clearest path to minimising uncertainties and advancing towards achieving the aims of genome‐wide genetic diversity assessments with enhanced comparability across studies and over time.

**FIGURE 1:**
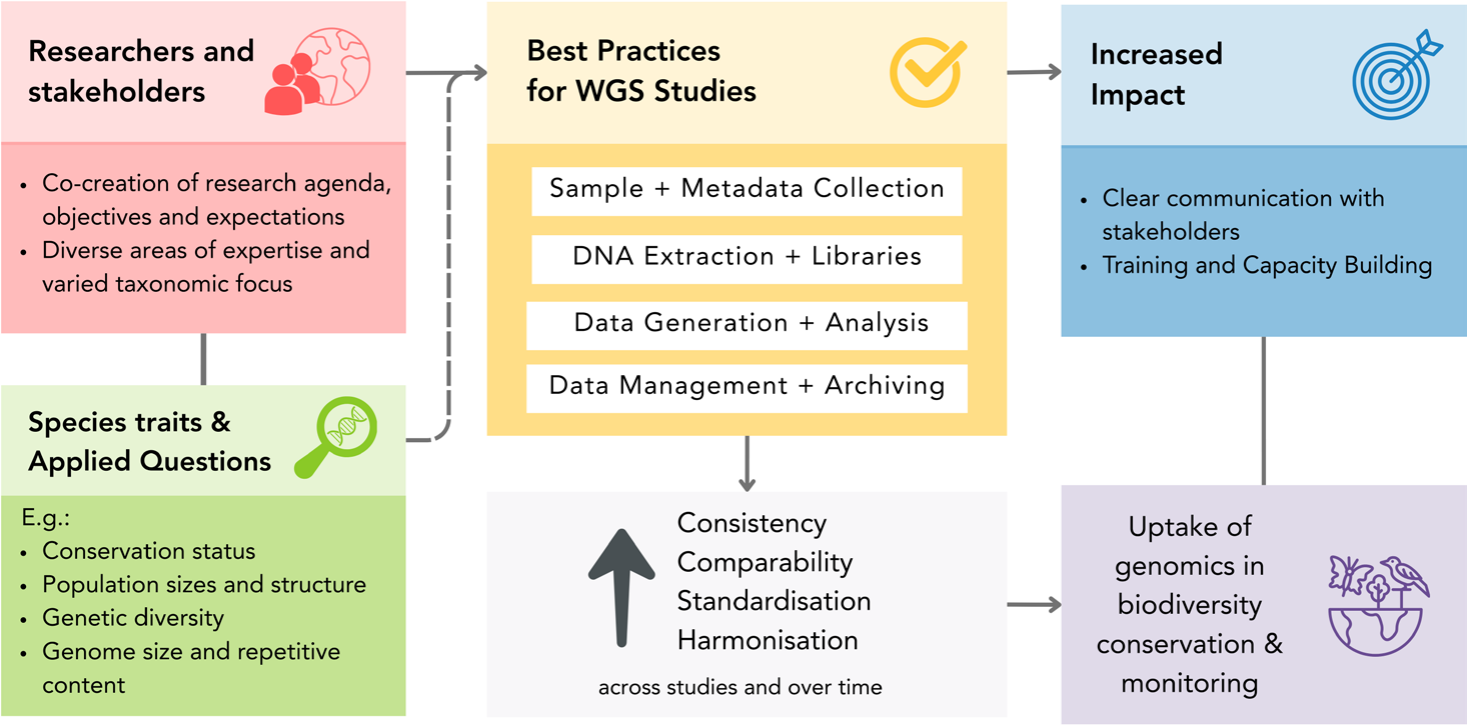
A framework for enhancing consistency and comparability of genetic diversity assessments through improved harmonisation and standardisation of key steps from study design to data archiving. Approaches employing whole genome re‐sequencing (WGS) data offer important opportunities for developing consensus standardisations enabling detailed genome‐wide analyses of genetic variation and more robust comparisons across studies and over time. In order to achieve increased impact it is essential for researchers to engage with stakeholders to co‐create research agenda that benefit from diverse areas of expertise considering the biology and ecology of the focal species and how genomic data integration will contribute to resolving the key questions to support conservation and monitoring. To fully realise the benefits offered by WGS data, methods and protocols that conform to common standards are needed to enhance confidence in the data produced, complemented by the standardised collection and management of contextual metadata. Comparability and reproducibility of genetic assessment studies also depends on the adoption of a consensus on analytical best practices, requiring the development of bioinformatics workflows that adhere to common sets of standards and agreeing on parameters and reporting formats for data processing and analysis. Community consensus on harmonised methods and standards in biodiversity genomics research practices leveraging reference genomes and WGS data will help to build a framework that enhances the uptake of genomics and better serves stakeholder needs in conservation.

To fully realise the benefits offered by WGS data, methods and protocols that conform to common standards are needed to enhance confidence in the data produced, similar to the progress that has been made with reference genomes. These methodological harmonisations form a key part of the larger framework for enhancing consistency and comparability of genetic diversity assessments through improved standardisations of key steps from study design to data archiving (Figure 1). Building consensus on harmonised approaches to WGS data production requires collaboration and input from researchers with experience of diverse study systems. Key points to begin to address will include aspects of study design, such as establishing recommended minimum sample sizes, e.g., at least 10 individuals per population (and sex where necessary) and best practices for temporal monitoring taking into account species life histories. Others will include technical specifications, such as defining the necessary sequencing coverage based on the techniques employed, e.g., a minimum coverage of 20–30× for hcWGS data. Further considerations for building a consistent framework would need to examine how data production recommendations might be tailored to best serve particular research questions and practical issues (e.g., large genomes with a high proportion of repetitive elements). For instance, while lcWGS might be suitable for specific population‐level studies where individual genotyping is less critical, hcWGS would be necessary for detecting rare variants, characterising structural variants (possibly coupled with long‐read data), or studying selection signatures. Community‐accepted best practices for data production would greatly improve consistency and comparability across studies and over time. However, realising this would additionally require addressing aspects of data management and archiving, such as defining practical mechanisms that promote adherence to FAIR Principles (Findable, Accessible, Interoperable, and Reusable) (see Glossary) (Leigh et al. 2024; Wilkinson et al. 2016). Here consensus‐building will need to focus on establishing minimum metadata requirements for population genomics studies, such as detailing sample collection information, akin to metadata standardisation efforts for reference genomes (Böhne et al. 2024). The management of contextual metadata is especially important for biobanking, which has an important role to play in long‐term genetic monitoring studies by enabling the storage of samples and associated data for future analyses. We therefore call upon the research community to consider the key questions that need addressing to achieve enhanced harmonisation of WGS data production and management.

Beyond production methods and protocols, the comparability and reproducibility of genetic assessment studies also depend on the adoption of a consensus on analytical best practices. This will require developing bioinformatics workflows that adhere to common sets of standards and agreeing on parameters and reporting formats for data processing and analysis. Community adoption of existing data science solutions offered by management systems such as Nextflow (di Tommaso et al. 2017) or Snakemake (Mölder et al. 2021) would support these aims. This could also be extended to considering how best to embrace advances in machine learning and artificial intelligence (AI) to handle high‐dimensional genomic data effectively (Korfmann et al. 2023; Schrider and Kern 2018). Importantly, achieving consensus and widespread uptake will require support through training on how to implement these analytical best practices, e.g., through platforms like the Australian Threatened Species Initiative (TSI) (see Glossary) Applied Conservation Genomics Hub (TSI Applied Conservation Genomics Hub 2024) and the ERGA Knowledge Hub (ERGA Knowledge Hub 2024). We therefore call upon the research community to examine the key topics that need addressing to achieve enhanced harmonisation of WGS data analysis methods.

### 2.4 Engagement With Stakeholders is Key to Achieving Meaningful Genomic Data Integration

Achieving community consensus on data production, management, and analysis methods for genome‐wide genetic diversity assessments is essential for the integration and widespread deployment of genomic data in biodiversity conservation and monitoring. While researchers have an important role to play, especially on the methodological and technological side, to fully succeed it is also essential to engage with stakeholders to co‐develop pathways to achieve meaningful genomic data integration (Bateman and Balmford 2023; Sandström et al. 2019; Supple and Shapiro 2018; Taylor et al. 2017). Here, stakeholders are people or organisations who affect or are affected by biodiversity conservation and management decisions. These may include policymakers, conservationists, natural site managers, non‐governmental organisations, landowners, farmers, environmental agencies, citizen scientists, local communities, the general public, or other entities. Expanding and improving the scope of genomics research in biodiversity conservation requires the involvement of relevant stakeholders and rights holders from the outset (Aitken et al. 2024; Leventon et al. 2016; Segelbacher et al. 2022; Supple and Shapiro 2018). By engaging local communities, policymakers, and conservation practitioners in a cooperative and transparent manner, researchers can ensure that scientific research and objectives are aligned with broader socio‐economic interests and ecological priorities (Reed 2008). Such inclusive, co‐created approaches help build trust, promote a sense of ownership, and strengthen accountability, all of which contribute to more effective and sustainable conservation outcomes (Skarlatidou et al. 2019; Sterling et al. 2017). By involving stakeholders early on, these efforts ensure that emerging genomic data standards and best practices reflect diverse perspectives, address practical challenges, and ultimately enjoy broader acceptance and lasting relevance in applied biodiversity conservation contexts. To begin to examine potential pathways for co‐development, we explored the landscape of stakeholder engagement with applied genomics research based on a set of case studies, a stakeholder identification survey, and a workshop of researchers for mapping stakeholders involved in biodiversity conservation research projects from several European countries.

Three ongoing case studies, spanning fungal diversity, butterfly cryptic variation, and aspen adaptation, were examined to identify practical examples of the effectiveness and value of integrating WGS data and reference genomes with meaningful stakeholder engagement (Box 1). Each project integrates genomics methodologies to overcome technical and conceptual hurdles, producing high‐resolution data that resolve otherwise intractable questions as well as informing conservation policies, Red List updates, and management decisions. Additionally, stakeholders, from citizen scientists and local agencies to a national tree breeding institute, play active roles in defining research questions, assisting in sample collection, interpreting results, and advocating for the adoption of genomic approaches, often directly requesting their implementation. Their involvement creates collaborations, addresses practical challenges, and ensures knowledge obtained through the application of genomic data is aligned with real‐world conservation needs. Collectively, these case studies provide examples illustrating how adopting WGS workflows in combination with stakeholder participation and decision‐making can lead to credible, context‐specific, and forward‐looking solutions. They emphasise the benefits of aligning standardisation efforts with stakeholder perspectives and highlight how co‐creation as collaborative activities during which multiple interdependent external stakeholders contribute to agreeing on the consensus approaches, can drive the widespread acceptance and applicability of harmonised genomic data.

BOX 1 **Three Case Studies on Stakeholder Engagement in Biodiversity Genomics.**

The summaries of three ongoing research projects highlight key points identified from the experiences of the interactions between biodiversity conservation research projects and a variety of stakeholders, with a research focus on fungi, butterflies and aspen trees.

**Genomic Insights for Fungal Conservation**

Project goals and methodology: Sweden's forests host approximately 10,400 fungal species (Knutsson et al. 2020), yet their genetic diversity remains largely unexplored and rarely integrated into conservation planning. To bridge this gap, this project combines reference genome assemblies and WGS data to characterise population structure and inform Red List assessments for three focal fungal species. Methodological decisions on sequencing and analysis approaches were informed by the researchers' experience working with arbuscular mycorrhizal fungi (Manyara et al. 2024; Sahraei et al. 2022). Stakeholder involvement includes the Swedish Species Information Centre and county administrations who guided species selection and sampling design, as well as citizen scientists who contributed with field‐collected samples to achieve broad geographical coverage.

Importance of genomics and stakeholder engagement: Producing and employing near‐chromosome‐level reference genomes and WGS data for approximately 100 samples per species, the study is developing a comprehensive first view of fungal genome‐wide genetic diversity and population structure, for protected species for which only distribution/occurrence data were previously known. Stakeholders initiated the research questions, with the goals of testing the applicability of genomics data approaches to deliver results that could help inform Swedish Red List updates and species action programmes, thereby creating from the outset a link between the project results and policy decisions. Engaging with county boards and citizen scientists enabled not only comprehensive sampling but also the incorporation of local knowledge in the study programme, and is proving an effective platform to improve communications on the benefits of genomics approaches to biodiversity conservation. This case study is illustrating how projects using genome‐wide genomics data can successfully engage with different stakeholders, here to collectively work towards actionable insights for fungi that represent a traditionally understudied kingdom in conservation genomics (May et al. 2019).

**Genomic Research on Swiss Butterflies**

Project goals and methodology: Switzerland's ~ 200 butterfly species present a tapestry of cryptic variation that conventional genetic approaches, such as DNA barcoding, microsatellites, or RADseq, have struggled to resolve (Jospin et al. 2023; Litman et al. 2018). This project is employing WGS data and chromosome‐level reference genomes to unravel the relationships within seven species complexes, aiming for a dozen individuals per taxon and sequencing hundreds of individuals so far. Population genomics analyses are standardised across species and aim to explore patterns of gene flow and contrast how some complexes represent distinct taxa while others reflect cases of subtle intraspecific diversification. Stakeholders include hobbyists, museums, consultants, and cantonal offices, who provided valuable field knowledge, brought together as part of efforts to update the book of Swiss butterflies (Schweizerischer Bund für Naturschutz 1994) coordinated by a community‐based not‐for‐profit society of Swiss lepidopterists.

Importance of genomics and stakeholder engagement: As traditional barcoding approaches often lack the resolution needed to clarify cryptic diversity, the study is focussed on employing WGS data for genetic diversity assessments. Stakeholders approached the researchers expressing their needs for a high‐resolution genomics solution to overcome challenges associated with studying and monitoring species complexes in the context of conservation programmes. By engaging with stakeholders the study is able to integrate key ecological knowledge of the species and their habitats with insights from the genomics data. An important consideration was to develop a strategy where the data and results could be comparable across the different species studied, and be “future‐proof” for reuse in follow‐up studies. This case study exemplifies how standardised WGS approaches can support conservation stakeholder decision‐making as the findings will be used to inform upcoming Red List revisions in Switzerland. Initial results highlight a spectrum of diversity amongst cryptic species, ranging from distinct taxa to intraspecific diversity and postglacial lineages, but also demonstrate that generally further studies are needed beyond a national scale, e.g. for the *Phengaris alcon*/*P. rebeli* complex (Lucek et al. 2024).

**Genomic Insights Into Aspen Adaptation**

Project goals and methodology: Aspen ( *Populus tremula* ) is a keystone poplar species supporting diverse insect communities (Robinson et al. 2012) and delivering vital ecosystem services across Scandinavia. To determine whether aspen populations have genetic adaptations to varying environmental conditions, the project is employing WGS data with a chromosome‐level reference genome assembly generated using multiple sequencing technologies (PacBio and Illumina, optical and genetic mapping) (Lin et al. 2018; Robinson et al. 2024). Geographic transects have previously enabled the identification of genetic variants linked to adaptive traits using WGS data (Wang et al. 2018), informing predictions about how these trees may respond to climate shifts (Ingvarsson and Bernhardsson 2020). The primary stakeholder is the Forestry Research Institute (Skogforsk), which is responsible for forest tree breeding programmes in Sweden to provide the sector with future‐resilient trees and ensure that within species genetic diversity and key genetic strains are maintained.

Importance of genomics and stakeholder engagement: Initially pursued in the context of basic research on understanding the adaptive potential of aspen trees to future climate conditions, the researchers first concentrated on generating a high‐quality reference genome assembly. While aspen has limited economic value for the forestry sector, engaging with Skogforsk identified common goals regarding the promotion of sustainably managed forests that reduce environmental damage and help to protect and restore biodiversity as part of their institutional mandate. Sampling strategies were informed by Skogforsk's field expertise to define transects running from north‐to‐south and east‐to‐west with Skogforsk personnel collecting samples and establishing two common garden trials. The partnership also means that a long‐term temporal strategy could be developed with the institute's help to implement and maintain the field trials comprising all genotypes being resequenced as part of the research. This case study demonstrates how stakeholder participation complements scientific research relying on genome‐wide data, while also ensuring that findings can be directly applied to biodiversity conservation and management strategies.

When the ERGA Citizen Science Committee (CSC) initiated its stakeholder identification survey in 2022, it became apparent that researchers in biodiversity genomics are often unaware of their potential stakeholders who could benefit from, be affected by, or play a role in the application of their scientific results. This impedes the establishment of effective relationships and hinders the ability to integrate stakeholder perspectives into research design, implementation, and future planning. Notably, some scientists perceived stakeholder participation requirements as an added burden rather than an opportunity. The results of the ERGA CSC survey thus indicate that the ERGA community, which represents a significant portion of the biodiversity genomics scientific community in Europe, requires guidance and training on how to identify, engage, and communicate with stakeholders in their work. Tools such as those outlined in the BiodivERsA Stakeholder Engagement Handbook (Durham et al. 2014) provide an excellent starting point for researchers to recognise and appreciate the value of diverse stakeholder views, enabling them to incorporate these perspectives more systematically into their work. To initiate the identification of pathways for co‐development, the ERGA CSC, in collaboration with the ERGA Pilot Project (Mc Cartney et al. 2024) and members of the Biodiversity Genomics Europe (BGE) Project (see Glossary), organised a workshop aimed at strengthening researchers' capacities to recognise relevant stakeholders.

**FIGURE 2:**
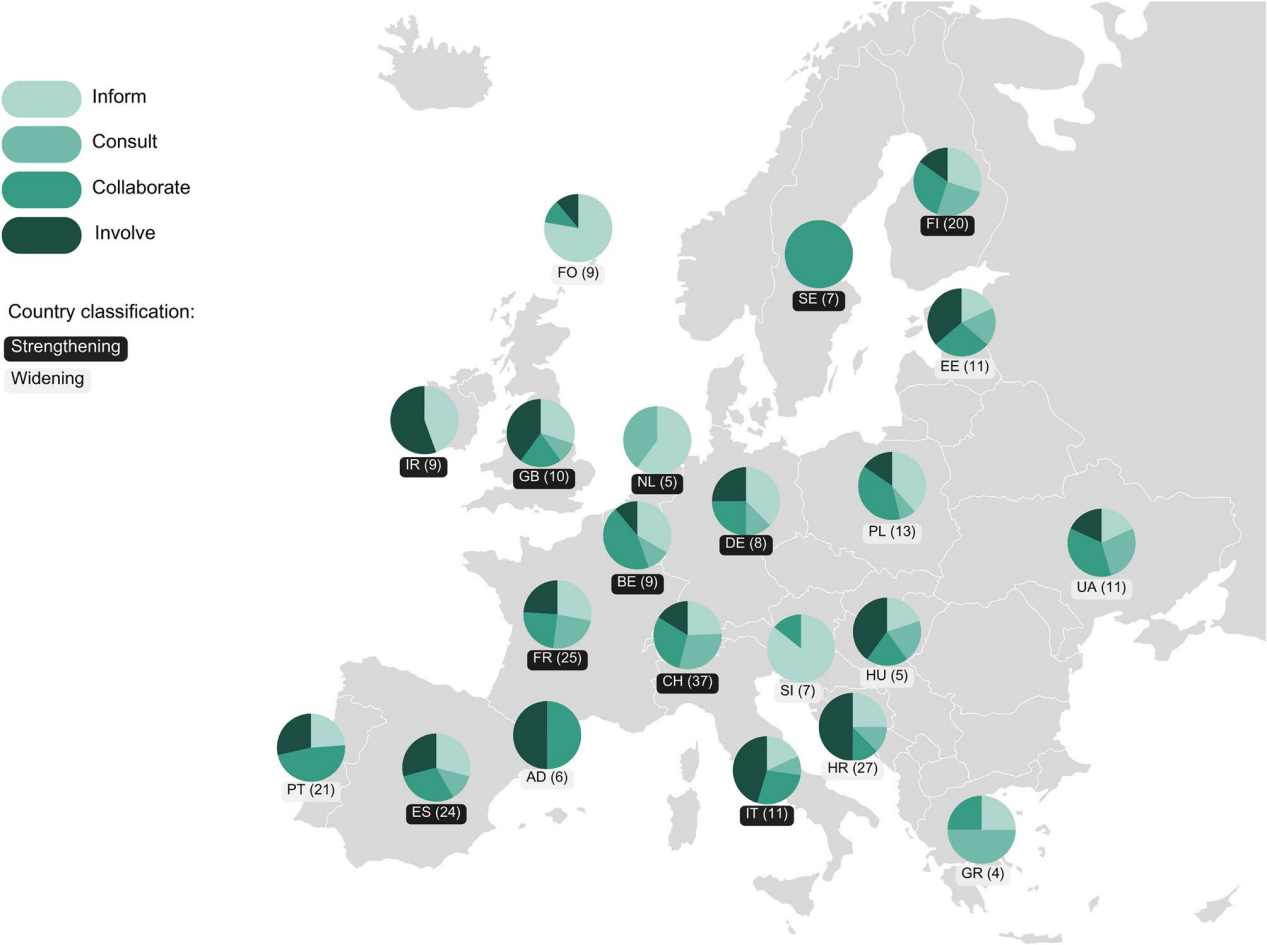
Proportions of the 283 identified stakeholders assigned to stage categories of influence and interest for 21 European countries. This exercise aimed to encourage the participating researchers to consider not only what categories of stakeholders and stakeholder groups might potentially be interested in their research projects, but also to begin to consider how engagement priorities and strategies could differ depending on the perceived influence and interest levels of different stakeholders. The categories and the stakeholder identification stages are described in the BiodivERsA Stakeholder Engagement Handbook (Durham et al. 2014): Inform (low influence and low interest—monitor these stakeholders and keep them regularly updated as needed, tailoring communications to meet their needs), Consult (high interest and low influence—provide these stakeholders with enough information and interaction to keep them updated and address their concerns, but avoid overwhelming them with too much information), Collaborate (high influence and high interest—essential stakeholders that must be fully engaged, enlist their full help, create partnerships, galvanise support for the project, and keep them satisfied), and Involve (high influence and low interest—keep these stakeholders adequately informed and maintain regular contact to ensure no major issues arise). Across all countries the categories with the most stakeholders were Inform with 85 stakeholders and Collaborate with 84, followed by Involve with 71 and Consult with 43 stakeholders. Countries are labelled using their International Organisation for Standardisation (ISO) 3166–1 alpha‐2 codes: Andorra (AD), Belgium (BE), Croatia (HR), Estonia (EE), Faroe Islands (FO), Finland (FI), France (FR), Germany (DE), Great Britain (GB), Greece (GR), Hungary (HU), Ireland (IR), Italy (IT), Netherlands (NL), Poland (PL), Portugal (PT), Slovenia (SI), Spain (ES), Sweden (SE), Switzerland (CH) and Ukraine (UA). The total number of stakeholders mapped for each country is shown in parenthesis. Stakeholders from Luxembourg and Romania were also present in the dataset, but are not shown in the figure due to low sample size (*N* = 2 for each).

The online workshop involved 39 researchers with a broad geographical representation covering 23 European countries, including both those classed as Strengthening or Widening Countries by the European Union (see Glossary) based on higher or lower performance in European research and innovation indicators. The goals were to identify, prioritise, and map international, national, and local stakeholders involved in or potentially interested in their biodiversity genomics research. Groups of participants identified potential stakeholders in biodiversity genomics and then considered how to prioritise them (Durham et al. 2014). The process of interactively grouping stakeholders based on their perceived influence and interest levels into four categories (Inform, Consult, Collaborate, and Involve) stimulated discussions that prompted participants to evaluate how they recognise and prioritise relevant stakeholders. Across all identified national‐level stakeholders, the largest groups were Inform (30%) and Collaborate (30%) and the smallest group was Consult (15%), with Involve accounting for 25% (Figure 2). There was no statistically significant association between country status (strengthening or widening) and stakeholder category, and there were no statistically significant differences between strengthening and widening Countries for any of the four categories (assessed using a chi‐squared test of independence and pairwise two‐proportion *z*‐tests, respectively). This suggests that despite the heterogeneity across countries, performance in research and innovation indicators is not strongly linked to how researchers perceive the influence and interest levels of different stakeholders. Discussions during the categorisation process revealed that stakeholders could be grouped into different categories depending on their current engagement in research projects. This fluidity, where stakeholders may transition between categories as projects generate more tangible results, highlights the dynamic nature of engagement.

Another important point that was raised repeatedly with respect to assessing a stakeholder's interest level was the highly variable perception of the value and utility of reference genomes and WGS data. Participants described how they noted a growing positive consideration of the importance of genetic diversity assessments. However, they also recognised a disconnect between this and the perceived relevance of genome‐wide genetic diversity assessments amongst practitioners. Taken together, these quantitative and qualitative observations suggest a need for additional efforts that aim to improve researcher‐stakeholder interactions. On the one hand, researcher awareness of and capacity to recognise relevant stakeholders in biodiversity genomics is an area that would benefit from further development. On the other hand, a demystification of genomics‐based approaches to applied conservation and monitoring would help foster wider and more meaningful stakeholder engagement. In this regard, developing and promoting community‐consensus‐validated genomics methodologies for assessing genome‐wide genetic diversity could substantially advance the integration of genomic information in the conservation and management of biodiversity and ecosystem services.

These initial efforts established by the ERGA CSC form part of a conceptual framework aimed at establishing best practices for stakeholder engagement in biodiversity genomics and contribute to closing the conservation genetics gap—the disconnect between scientific knowledge of the utility of genetic data and their effective application in conservation management (Heuertz et al. 2023; Klütsch and Laikre 2021; Sandström et al. 2019). In the next phase, the ERGA CSC will conduct interviews with stakeholders from various sectors, including politics, industry, and local communities, to gain deeper insights into their motivations, concerns, and expectations when invited to participate in scientific studies on biodiversity genomics. Ultimately, these approaches aim to develop a long‐term plan for meaningful and equitable engagement, ensuring that genomics research activities not only adhere to scientific standards but also resonate with the priorities and realities of those most affected by and invested in biodiversity conservation.

## 3 Achieving Standardisation in Applied Research Requires Overcoming Barriers

Despite the many opportunities, integrating genomic information into conservation efforts faces substantial challenges. Even as the scientific community defines and refines standards, additional barriers must be addressed to ensure global, equitable, and actionable best practices. These include sequencing costs and automatisation, the need for specialised expertise, difficulties interpreting and communicating results to stakeholders, and a lack of standardised procedures (Cook et al. 2023; Hogg 2024; Kardos et al. 2021; Klütsch and Laikre 2021; Shafer et al. 2015). Even when feasible, the heterogeneity across various genetic and genomic approaches impacts the practicality of broadly implementing DNA‐based methods (DeWoody et al. 2021; Hoban, Bruford, et al. 2023; Hogg 2024). Moreover, pursuing high standards for accuracy and comparability without a plan for integration of diverse nations could widen the feasibility gap in resource‐limited regions, such as South America, Africa, and Southeast Asia (Linck and Cadena 2024; Wilson et al. 2016). Variations in priorities and preferred approaches amongst scientists and stakeholders to understand biodiversity can further complicate genomic data integration (Grill 2021; Klütsch and Laikre 2021; Taylor et al. 2017). However, given the rapid technological advances and the ongoing efforts to integrate genomics into biodiversity management, prioritising complementary actions that address these challenges and facilitate the incorporation of genomic data into evidence‐based decision‐making is paramount (Hogg 2024).

### 3.1 Access to Genomic Data Needs to Be More Evenly Distributed

A primary obstacle to implementing harmonised methods in genome‐wide genetic diversity assessments is the uneven distribution of access to genomic data generation and downstream analytical computational infrastructure worldwide (Linck and Cadena 2024; Pearman et al. 2024). Many regions, particularly those with limited financial resources and dedicated infrastructures, are unable to generate comprehensive genomic datasets, leading to an incomplete understanding of global biodiversity. This partial understanding is also due to a tendency to focus on well‐known parts of the genome where we already have established expectations, such as specific genetic markers or transcribed genic regions (Theissinger et al. 2023). This imbalance could be partially addressed by enhancing open access practices and archiving of datasets with contextual metadata (Leigh et al. 2024). However, merely sharing data is insufficient if reporting practices, including metadata standards, do not fulfil minimum standards requirements, as this makes data use and reuse more challenging. To address these disparities, international collaborations and funding initiatives must support local capacity to produce, archive and analyse data. The open science movement for making scientific research and data openly accessible is helping to address some inequities, where faster and broader adoption will improve access to genomic data (Winker et al. 2023). For example, requirements to archive datasets at public repositories like the European Nucleotide Archive (Yuan et al. 2024) are a key first step towards improved access. Advancing consistent (meta)data standards while improving equitable access will allow a more diverse range of contributors to shape the global harmonisation process.

### 3.2 Broadening Participation by Reducing Economic and Logistical Barriers

The costs, logistics complexities, and infrastructure needs associated with genome‐wide data production and analyses still limit the integration of fully standardised approaches, especially in resource‐limited settings (Bertola et al. 2024). Although there are always trade‐offs to consider when financing conservation actions, sequencing costs continue to decrease and partnerships that increase data‐generation throughput can further reduce per‐base expenses. Beyond costs associated with sequencing, expertise, equipment and reliable computing resources often remain challenging to obtain. Reducing these barriers involves promoting broadly accessible cloud computing solutions, developing and disseminating free‐to‐use data analysis workflows, and supporting reproducible analysis pipelines through open access platforms (di Tommaso et al. 2017; Larivière et al. 2024; Mölder et al. 2021; The Galaxy Community et al. 2024). By making (bio)informatics tools and workflows readily available and easy to use through clear documentation and tutorials, researchers and practitioners will be more willing to adopt these best practices. Such inclusionary measures encourage global standards uptake, ensuring that fully standards‐compliant methods do not remain an exclusive domain of well‐resourced research groups or countries.

### 3.3 Strengthening Knowledge Transfer and Capacity Building

Insufficient training opportunities and limited knowledge transfer activities impede the global adoption of harmonised genomics applications. Conservation research is often misaligned with areas of highest biodiversity, partly because local scientists and practitioners in these regions lack the required training, infrastructure, and technical and analytical support (di Marco et al. 2017; Schiebelhut et al. 2024). To remedy this, community‐driven initiatives must focus on delivering accessible training, developing open educational material and resources, and fostering long‐term collaborative networks. Examples include the African BioGenome Project's (see Glossary) distributed regional workshops (Sharaf et al. 2024), the ERGA Knowledge Hub's community‐curated training materials (ERGA Knowledge Hub 2024), and the Australian TSI's online toolkit (Hogg et al. 2024). By empowering local researchers and practitioners with the skills, knowledge, and capacity to produce and interpret genomic data locally, it becomes more feasible to implement and maintain agreed‐upon standards.

### 3.4 Communication Needs Strengthening to Maximise Impact and Reach

Clear, transparent communication is essential to ensure that stakeholders, policymakers, citizen scientists, and the general public fully appreciate the benefits and limitations of genomic approaches to biodiversity conservation (Garner et al. 2016; Kadykalo et al. 2020). Without effective communication, misunderstandings may emerge—for instance, confusing species‐level identification from DNA barcoding or community‐level characterisations via metabarcoding with the more detailed genetic diversity insights provided by WGS data (Couton et al. 2023). Such misconceptions have the risk of generating unrealistic expectations and may discourage the adoption of standardised methods, particularly by stakeholders. Enhancing communication strategies, collaborating with professional science communicators, and presenting information in accessible formats for non‐specialists can foster trust (e.g., avoiding or ‘translating’ jargon terminology), clarify the potential and limitations of each genomics‐based tool, and support the incorporation of consensus standards into practical conservation and monitoring initiatives.

### 3.5 Trust and Confidence Are Needed for Widespread Adoption of Genomics Tools

Achieving global standardisation requires that scientists and stakeholders have trust in the data, methods, and outcomes of genomics‐based assessments. Ensuring adherence to FAIR principles for data management (Wilkinson et al. 2016) and, where appropriate, CARE Principles (see Glossary) (Carroll et al. 2020), as well as recognising the tenet of making data “as open as possible and as closed as necessary”, can help build this trust, especially for data under ethical considerations. Equally important is the ability to engage with stakeholders in their local languages and cultural contexts, aided by AI‐driven translation tools that can help break down communication barriers. Demonstrating that the scientific community is converging on consensus standards for genome‐wide genetic diversity assessments instils confidence in end‐users, encouraging them to move beyond familiar but less informative techniques. By highlighting tangible conservation benefits, practitioners and other stakeholders can appreciate the added value of employing harmonised, genome‐wide approaches, ultimately accelerating the adoption of these methods and advancing global conservation efforts.

## 4 Discussion

Community consensus on harmonised methods and standards in biodiversity genomics research practices leveraging reference genomes and WGS data will help to build a framework that better serves stakeholders' needs in conservation. The way forward to guide the formulation of this commonly agreed‐upon set of standardised best practices will need to take into account three principal considerations: (i) researcher‐focused methodological and technological harmonisations; (ii) inclusivity‐aware accessibility and participation solutions; and (iii) stakeholder‐aimed practical and mutually beneficial partnerships. Practical next steps should likely cover complementary dimensions: on the one hand, continuing to develop scientific use cases, demonstrating how enhanced comparability can be achieved, and on the other hand, extending initiatives exploring researchers' experiences and perceptions of interactions with stakeholders and vice versa. As set out above, the advantages offered by using reference genomes and genome‐wide data for genetic diversity assessments provide compelling arguments for the widespread integration of such genomic information into biodiversity conservation and genetic monitoring applications. However, this comes with several challenges, particularly with respect to overcoming barriers to the adoption and implementation of uniform practices worldwide. Nevertheless, opportunities for the research community to start to build consensus on data production, management, and analysis methods for genome‐wide assessments must be seized. This initiative can build on achievements in harmonising best practices for reference genome generation to achieve the highest possible levels of accuracy, interpretability, and comparability. It will also require engagement with stakeholders to co‐develop pathways for the meaningful integration of genomic data, as well as alignment with ongoing global efforts defining standardised information to measure and report genetic diversity. Demonstrating that researchers are committed to converging on these best practices is a key first step, starting here by explaining the rationale and highlighting likely challenges and possible solutions in order to galvanise support across the community.

**Glossary**
African BioGenome Project: This initiative is a coordinated pan‐African effort to build capacity and infrastructure to generate, analyse, and deploy genomic data for the improvement and sustainable use of biodiversity and agriculture across Africa (Ebenezer et al. 2022)
Biodiversity Genomics Europe (BGE) Project: This project is a joint effort of two genomics communities: iBOL Europe, which focuses on DNA barcoding, and the European Reference Genome Atlas (ERGA), which focuses on genome sequencing, collectively aiming to accelerate the use of genomics science to enhance understanding of biodiversity (https://biodiversitygenomics.eu/)
California Conservation Genomics Project: A state‐funded programme to generate, analyse, and curate a high‐quality reference genome and 100–150 resequenced genomes for species that span the ecological and phylogenetic breadth of California's marine, freshwater, and terrestrial ecosystems (Shaffer et al. 2022)
CARE Principles: The acronym stands for Collective Benefit, Authority to Control, Responsibility, and Ethics, a set of principles aiming to address concerns related to protecting the rights and interests of people and the purpose of data to ensure equitable outcomes (Carroll et al. 2020)
Earth BioGenome Project (EBP): An international initiative that aims to coordinate global efforts to sequence, catalogue, and characterise reference‐quality genomes of all of Earth's eukaryotic biodiversity (Lewin et al. 2018)
Effective population size (*N*_e_): In population genetics, *Ne* is defined as the size of an ideal population that experiences the same amount of genetic drift and increase of inbreeding as the real population (Wright 1931). *Ne* is one of the most important parameters for assessing the long‐term viability of species, and its values are often far lower than the number of individuals (census size, *Nc*) (Fedorca et al. 2024). It reflects the genetic diversity and evolutionary potential of a population, accounting for factors like unequal sex ratios, variation in reproductive success, and changes in population size over time. This measure is crucial in determining the level of variability in a population and the effectiveness of selection relative to drift (Charlesworth 2009)
European Reference Genome Atlas (ERGA): A pan‐European scientific community of experts in genome sequencing and analysis that aims to coordinate the generation of reference‐quality genomes for all eukaryotic species in Europe (Mazzoni et al. 2023)
FAIR Principles: The acronym stands for Findability, Accessibility, Interoperability, and Reuse, a set of principles used to assess the capacity of computational systems to find, access, interoperate, and reuse data with no or minimal human intervention (Wilkinson et al. 2016)
Genome‐wide genetic diversity: The complete diversity (or genetic variation) found across the entire genome of one individual of a species, encompassing all types of variations, including SNPs, insertions and deletions (indels), copy number variations, and structural variants
Reference genome: The digital representation of the complete DNA sequence of a species, typically obtained from assembling contiguous sequences produced by long‐read technologies into complete, high‐quality chromosome‐level genome assemblies (Li and Durbin 2024)
Runs of homozygosity (RoH): The contiguous homozygous regions in an individual's genome that can be identified as identical haplotypes inherited from each parent, used to inform measures of inbreeding where recent inbreeding results in long runs of homozygosity
Standardisation and harmonisation: Although often used interchangeably, these are distinct terms that refer to efforts to achieve or enhance uniformity and comparability across different approaches, respectively. Standardised approaches mean following agreed‐upon standards such as a particular method, format, or rule, while harmonised approaches mean making different systems compatible or aligned so that results can be more easily compared
Strengthening or Widening Countries: European Union member states and countries associated with the Horizon Europe Programme designated as well‐performing (strengthening) or under‐performing (widening) in research and innovation indicators, generally characterised by stronger research institutional frameworks and support (REA 2023)
Threatened Species Initiative (TSI): Australia's national project to improve conservation practices through the use of cutting‐edge genomics technology and advanced computational biology to transform the way the conservation industry manages wildlife recovery programmes (Hogg et al. 2022)
Whole‐genome re‐sequencing (WGS): The sequencing of an individual's entire genome content to identify genetic variation by comparing it to a reference genome, ranging from SNPs to insertions, deletions, and copy number variations (Bentley 2006)

## Author Contributions

C.G. and E.B. participated in the project conceptualisation and methodology and conducted stakeholder interviews with N.R.S., K.L. and A.R. C.G. also contributed to the formal analysis and data curation, while E.B. co‐led the supervision. C.J.M. played a role in supervision, conceptualisation, and methodology, and contributed to writing and editing the manuscript. A.M. helped with the investigation and contributed to the writing of the original draft and revisions. C.B., N.R.S., K.L., A.R., L.O., L.S.M. and M.J.R.‐L. drafted, reviewed, and revised the manuscript. J.M.‐F. and E.O. reviewed and revised the manuscript, with E.O. providing the perspective as a stakeholder. R.M.W. supervised the project, contributed to the conceptualisation, and coordinated editing inputs from all authors to produce the manuscript and revised manuscript.

## Disclosure

Benefit‐Sharing Statement: Benefits Generated: Benefits from this research accrue from the open sharing of our data and results as described above.

## Conflicts of Interest

The authors declare no conflicts of interest.

## Supporting information


Appendix S1.

## Data Availability

The data from the workshop are made available in Appendix S1.
